# Association between state unemployment rate and inpatient hospitalizations for tobacco use disorder

**DOI:** 10.18332/tpc/149482

**Published:** 2022-05-25

**Authors:** Elizabeth Wahlenmayer, Aparna Soni

**Affiliations:** 1 Peace and Violence Research Lab, School of Public Affairs, American University, Washington, United States; 2 Department of Public Administration and Policy, School of Public Affairs, American University, Washington, United States; 3 IZA Institute for Labor Economics, Bonn, Germany; 4 Center for Financial Security, University of Wisconsin-Madison, Madison, United States

**Keywords:** unemployment, hospitalizations, tobacco use disorder

**Dear Editor,**

Cigarette consumption increases during recessions^1-4^, but the downstream impact of unemployment on smoking-related health outcomes is less explored. This study examines the relationship between local unemployment rates and hospitalizations for tobacco use disorder (TUD). TUD is a clinical diagnosis of severe physiological dependence on tobacco. TUD does not necessarily indicate a long smoking history; even novice or occasional smokers can develop TUD. TUD poses severe health and social consequences beyond those associated with smoking alone, including clinically significant impairment or distress, recurrent use in physically hazardous situations, and failure to fulfill major role obligations^5^. In contrast with smokers who attempt to quit using outpatient therapies such as smoking cessation products, TUD patients are usually treated in inpatient settings, where they are removed from smoking cues and have access to pharmaceutical and behavioral treatment^5^.

In the US, there are nearly 4.5 million TUD hospitalizations per year nationwide, and hospitalizations nearly doubled from 713 per 100000 population in 2001 to 1415 in 2014. TUD hospitalizations pose significant costs on the healthcare system, with an average hospital charge of US$36830 per hospitalization; Medicare and Medicaid pay for more than half of these hospitalizations^6^. Meanwhile, unemployment reached record highs during the COVID-19 pandemic. In the current uncertain economic climate, it is critical to understand and address the economic determinants of TUD.

We explored this question using state-level data on inpatient hospitalizations from the 2001–2014 Healthcare Cost and Utilization Project (HCUP), which is representative of 38 states^6^. We identified inpatient hospitalizations with diagnostic code ICD-9 305.1 as those related to TUD. Hospitalizations were measured as the rate of TUD hospitalizations per 100000 population.

We supplemented HCUP with state-level data on unemployment rate, poverty rate, percent of population aged >65 years, and percent of non-White population from the US Census Bureau and Bureau of Labor Statistics^7^. We also included data on state-level cigarette excise taxes from the Tax Foundation^8^. Our sample size was 396 state-year observations.

We estimated the correlation between state unemployment rate and TUD hospitalizations using linear regression models with two-way fixed effects. Our model controlled for percent of state population above 65 years, percent of state non-White population, poverty rate, cigarette excise tax, state fixed effects, and year fixed effects. We used Stata version 16 to conduct all analyses.

Table 1 presents the regression results (more details in the Supplementary file). We found that a one percentage point increase in the state unemployment rate was associated with nearly 33 additional TUD inpatient hospitalizations per 100000 population (95% CI: 15.85, 50.08). This was equivalent to a 2.8% increase from baseline levels.

**Table 1:** Association between unemployment rate and rate of tobacco use disorder (TUD) hospitalizations (N=396)

*Variable*	*TUD hospitalizations^a^*
Unemployment rate	32.97***
	(15.85, 50.08)
Percent of population above 65 years	-54.65**
	(-91.70, -26.50)
Percent of population non-White	-59.10***
	(-91.69, -26.50)
Poverty rate	-13.70
	(-37.06, 9.66)
Cigarette excise tax	52.21***
	(18.33, 86.09)
R^2^	0.95
Mean of outcome variable	1168.64

a Mean rate of hospitalizations across all years, per 100000 population. Calculations based on data from HCUPNet, BLS, CDC, and Census Bureau 2001–2014. Table presents coefficient estimates and 95% confidence intervals in parentheses. The regression model also included state fixed effects and year fixed effects (results not shown). The outcome variable is the rate of tobacco use disorder hospitalizations (ICD-9 305.1; all-listed diagnoses) per 100000 population.

* p<0.1;

** p<0.05;

*** p<0.01.

In sensitivity analyses (available on request), our results were robust to the exclusion of control variables and controlling for indoor smoking bans. Heterogeneity tests indicate that the relationship between unemployment and increased TUD hospitalizations was driven by Whites.

While a large literature established a link between unemployment and cigarette consumption^1^, this is the first study to show downstream effects on TUD hospitalizations. Policymakers should be prepared for higher TUD rates during recessions and should take proactive measures to address tobacco-related disease during times of economic distress.

## Data Availability

The data supporting this research are available from the Healthcare Cost and Utilization Project database at https://hcupnet.ahrq.gov/
